# Supplementary material for: Radiomic features of axillary lymph nodes based on pharmacokinetic modeling DCE-MRI allow preoperative diagnosis of their metastatic status in breast cancer
Source: PLoS One. 2021 Mar 1;16(3):e0247074. doi: 10.1371/journal.pone.0247074 (PMC7920570; doi:10.1371/journal.pone.0247074)
Supplement: S1 Table — (DOC) [file pone.0247074.s001.doc]

| S1 Table. Texture features of T1 DCE-MRI in the study | | | | | |
| --- | --- | --- | --- | --- | --- |
| First-order statistical features | Histogram features | Gray-level co-occurrence matrix (GLCM) features | Haralick features | RLM features | MorphologyMetrics features |
| Min Intensity | Skewness | GlcmBinSize | HaraEntroy | Min Intensity2 | VolumeMM |
| Max Intensity | Kurtosis | GlcmTotalFrequency | AngularSecondMoment | Max Intensity2 | VolumeCC |
| Median Intensity | Uniformity | GlcmMatrixMean | Contrast | NumberOfIntensityBins | SurfaceArea |
| MeanValue | Energy | GlcmRelativeFrequency | HaraVariance | MinSize | SurfaceVolumeRatio |
| Std Deviation | Entropy | GlcmEnergy | SumAverage | MaxSize | Compactness1 |
| Variance | FrequencySize | GlcmEntropy | SumVariance | NumberOfSizeBins | Compactness2 |
| Volume Count | UPP | Inertia | SumEntropy | ShortRunEmphasis | Maximum3DDiameter |
| Voxel Value Sum | MPP | Correlation | DifferenceVariance | LongRunEmphasis | SphericalDisproportion |
| RMS | Quantile5 | InverseDifferenceMoment | DifferenceEntropy | GreyLevelNonuniformity | Sphericity |
| Range | Quantile10 | ClusterShade | InverseDifferenceMoment | RunLengthNonuniformity |  |
| Mean Deviation | Quantile25 | ClusterProminence |  | LowGreyLevelRunEmphasis |  |
| Relative Deviation | Quantile50 | HaralickCorrelation |  | HighGreyLevelRunEmphasis |  |
|  | Quantile75 | InvalidFeatureName |  | ShortRunLowGreyLevelEmphasis |  |
|  | Quantile90 |  |  | ShortRunHighGreyLevelEmphasis |  |
|  | Quantile95 |  |  | LongRunLowGreyLevelEmphasis |  |
|  |  |  |  | LongRunHighGreyLevelEmphasis |  |
