# Supplementary material for: Radiomic features of axillary lymph nodes based on pharmacokinetic modeling DCE-MRI allow preoperative diagnosis of their metastatic status in breast cancer
Source: PLoS One. 2021 Mar 1;16(3):e0247074. doi: 10.1371/journal.pone.0247074 (PMC7920570; doi:10.1371/journal.pone.0247074)
Supplement: Attachment — Submitted filename: 2 Response to Reviewers.docx [file pone.0247074.s002.docx]

**Response to the concerns raised by the reviewers (The First Part) and the academic editor (The Second Part)** (*quoted in italics and numbered for ease of reference*). All changes have been indicated red in the marked-up copy of revised manuscript labeled 'Revised Manuscript with Track Changes copy'.

**The First Part (Response to the comments raised by reviewers):**

*[Note: HTML markup is below. Please do not edit.]*

*Reviewers' comments:*

*Reviewer's Responses to Questions*

*Comments to the Author:*

*1. Is the manuscript technically sound, and do the data support the conclusions?*

*The manuscript must describe a technically sound piece of scientific research with data that supports the conclusions. Experiments must have been conducted rigorously, with appropriate controls, replication, and sample sizes. The conclusions must be drawn appropriately based on the data presented.*

*Reviewer #1: Yes*

*Reviewer #2: Yes*

*2. Has the statistical analysis been performed appropriately and rigorously?*

*Reviewer #1: Yes*

*Reviewer #2: Yes*

*3. Have the authors made all data underlying the findings in their manuscript fully available?*

*The PLOS Data policy requires authors to make all data underlying the findings described in their manuscript fully available without restriction, with rare exception (please refer to the Data Availability Statement in the manuscript PDF file). The data should be provided as part of the manuscript or its supporting information, or deposited to a public repository. For example, in addition to summary statistics, the data points behind means, medians and variance measures should be available. If there are restrictions on publicly sharing data—e.g. participant privacy or use of data from a third party—those must be specified.*

*Reviewer #1: Yes*

*Reviewer #2: Yes*

*4. Is the manuscript presented in an intelligible fashion and written in standard English?*

*PLOS ONE does not copyedit accepted manuscripts, so the language in submitted articles must be clear, correct, and unambiguous. Any typographical or grammatical errors should be corrected at revision, so please note any specific errors here.*

*Reviewer #1: Yes*

*Reviewer #2: Yes*

*5. Review Comments to the Author*

*Please use the space provided to explain your answers to the questions above. You may also include additional comments for the author, including concerns about dual publication, research ethics, or publication ethics. (Please upload your review as an attachment if it exceeds 20,000 characters)*

*Reviewer #1: This is a well written study to report the differentiation of 87 malignant and 89 benign axially lymph nodes seen in breast MRI, by using 4 different models: 1) conventional model based on image features; 2) PK-model based on pharmacokinetic parameters with their corresponding histogram features; 3) radiomic model based on all radiomic features, and 4) the combined model integrating radiomic features with conventional image features. The methods were pretty standard and well described. The results showed that the radiomics model based on texture had the best performance, and that was attributed to the* *heterogeneity in malignant compared to benign nodes. Although the results could achieve very high accuracy, it is still difficult to implement the complicate models in a clinical setting. Performing a careful comparison with the conventional image reading of the nodes to demonstrate how the radiomics model may help in the cases that the reading was wrong will greatly improve the value of this work. Some comments below:*

Response 1: We would like to thank the reviewer for the kind and helpful advice. This was a preliminary study in which we focused on the feasibility of use of radiomic features of axially lymph nodes in preoperative diagnosis of their metastatic status in breast cancer. In the study, we have used the *Delong* test to compare the overall diagnostic performance of different models. The result showed that the overall diagnostic performance of the **combined model and the radiomic model were significantly better than that of the pharmacokinetic model** and the conventional model (P<0.05). In clinical setting, it is really hard to demonstrate how the radiomics model may help in every single case in which the reading was wrong by conventional image feature. Following your helpful advice and based on this preliminary result, we will try to design another research with larger sample in order to achieve results that are more applicable in clinical settings.

*1. In the manuscript that I downloaded for review,* *Tables 1 and 2 were not included, thus it was difficult to compare the performance (sensitivity vs. specificity) between the radiomics and conventional models, which is*

*the most interesting part in my opinion.*

Response 1: We apologize for not showing the *Tables 1 and 2* in the original manuscript due to some technical problems. We have corrected the omission in the revised manuscript. Table 1 in original manuscript shows the texture features in the study. Table 2 shows the diagnostic performance (including the sensitivity and specificity) of four models for detection of metastatic axillary lymph nodes.

*2. There are well established nodal features commonly used to predict malignant vs. benign nodes, as described in this work, including the long and short axis length, short-long axis ratio, fatty hilum status, signal intensity on diffusion weighted imaging (DWI), and heterogenous enhancement feature. Please add a table to compare these features between the malignant and benign groups.*

Response 1: As suggested, we have added a new table (Table 1 in the revised manuscript) to compare the conventional image features between the malignant and benign groups.

*3. Please give the number of true positive, true negative, false positive, and false negative cases predicted by each of the 4 models. Then compare the results of reading and radiomics to see which cases were correctly predicted by radiomics but wrongly diagnosed by reading. Show these case examples using figures. These results will be very interesting.*

Response 1: Thank you for your insightful comment. In our research, the models constructed by training dataset and their overall performance were tested by the validation dataset. Thus, we cannot show which cases were correctly predicted by radiomics but wrongly diagnosed by conventional reading due to the very nature of radiomic analysis. It is hard to show which cases were correctly predicted by radiomics but wrongly diagnosed by conventional reading.

*4. The presence of fatty hilum is considered as an important benign feature, but it is not clear how this feature can be well appreciated on the DCE imaging sequence used in this study, that heavily focuses on a high temporal resolution (thus may sacrifice the spatial resolution). What other MR imaging sequences were considered in the reading? Please discuss.*

Response 1: The presence of fatty hilum was evaluated based on T2WI sequences. The MRI acquisition which has been described extensively in our previous study[1] were not specific to the current research. Therefore, we presented only a brief description of the MRI acquisition. We apologize for not clearly stating this before. We have made this clearer in the revised manuscript.

1.	Luo HB, Du MY, Liu YY, Wang M, Qing HM, Wen ZP, Xu GH, Zhou P, Ren J: **Differentiation between Luminal A and B Molecular Subtypes of Breast Cancer Using Pharmacokinetic Quantitative Parameters with Histogram and Texture Features on Preoperative Dynamic Contrast-Enhanced Magnetic Resonance Imaging**. *Acad Radiol* 2020, **27**(3):e35-e44.

*5. After adding results in issues 2-4, please also add discussion to elaborate the value of the radiomics that may be complementary to reading. In fact, it seems all 4 models have very high accuracy; therefore, the actual value of the very complicated models may be limited. Please emphasize on the discordant cases between reading and radiomics models.*

Response 1: As suggested, we have added the results and discussion in the revised manuscript to address this issue.

*6. Which DCE frame was used for drawing the nodal ROI? Please see issue 4) and show case example to illustrate that fatty hilum was included in the analysis.*

Response 1: The early stage of post-contrast image of DCE-MRI was used for drawing the whole nodal ROI. We are so sorry for not clearly stating this before, and we have made this clearer in the revised manuscript. Besides, the fatty hilum was evaluated on T2WI sequences (illustrated in our response to issue 4).

*7. How were the training and validation cases separated in the analysis, also in Figure 3 and Figure 5?*

Response 1: The training and validation cases were separated at a ratio of 7 to 3 in the analysis. We have added this information in the revised manuscript and in figure legends 3 and 5.

*Reviewer #2: I would like to thank the authors for a well written scientifically sound manuscript addressing an important topic which is being more and more explored in the current era. Hopefully more studies such as this one would help lead the community to perform bigger controlled trials and make the AI softwares vastly available in the clinic in the near future.*

Response 2: Thank you very much for your kind comments. We completely agree that it is important to judge the metastatic status of lymph nodes, especially in patients with breast cancer. In clinical settings, it is really difficult to fulfill this task by conventional image features. Radiomics is a novel and prospective way to assess the tumor heterogeneity which may be the unique characteristic of malignant tumors. This was our motivation to perform this study. However, this was a preliminary research and our results need to be verified in larger multi-center studies. The results demonstrate the feasibility of utilization of radiomic features of axillary lymph nodes for diagnosing their metastatic status in patients with breast cancer.

*6. PLOS authors have the option to publish the peer review history of their article (what does this mean?). If published, this will include your full peer review and any attached files.*

*If you choose “no”, your identity will remain anonymous but your review may still be made public.*

*Do you want your identity to be public for this peer review? For information about this choice, including consent withdrawal, please see our Privacy Policy.*

*Reviewer #1: No*

*Reviewer #2: No*

**The Second Part (Response to the points raised by the academic editor):**

*Journal Requirements:* *When submitting your revision, we need you to address these additional requirements.*

*1. Please ensure that your manuscript meets PLOS ONE's style requirements, including those for file naming. The PLOS ONE style templates can be found at*

*https://journals.plos.org/plosone/s/file?id=wjVg/PLOSOne_formatting_sample_main_body.pdf and*

*https://journals.plos.org/plosone/s/file?id=ba62/PLOSOne_formatting_sample_title_authors_affiliations.pdf*

Response 3: We have revised our manuscript in accordance with the additional requirements of PLOS ONE.

*2. We suggest you thoroughly copyedit your manuscript for language usage, spelling, and grammar. If you do not know anyone who can help you do this, you may wish to consider employing a professional scientific editing service.*

*Whilst you may use any professional scientific editing service of your choice, PLOS has partnered with both American Journal Experts (AJE) and Editage to provide discounted services to PLOS authors. Both organizations have experience helping authors meet PLOS guidelines and can provide language editing, translation, manuscript formatting, and figure formatting to ensure your manuscript meets our submission guidelines. To take advantage of our partnership with AJE, visit the AJE website (http://learn.aje.com/plos/) for a 15% discount off AJE services. To take advantage of our partnership with Editage, visit the Editage website (www.editage.com) and enter referral code PLOSEDIT for a 15% discount off Editage services. If the PLOS editorial team finds any language issues in text that either AJE or Editage has edited, the service provider will re-edit the text for free.*

*Upon resubmission, please provide the following:*

1) *The name of the colleague or the details of the professional service that edited your manuscript*
2) *A copy of your manuscript showing your changes by either highlighting them or using track changes (uploaded as a *supporting information* file)*
3) *A clean copy of the edited manuscript (uploaded as the new *manuscript* file)*

Response 3: As suggested, the manuscript and the revised manuscript have been edited and proofread by the *Medjaden* Bioscience Limited, a professional medical editing company. A copy of our revised manuscript showing the changes using track changes has been uploaded as a *supporting information* file.

*3. We note that you have indicated that data from this study are available* *upon request. PLOS only allows data to be available upon request if there are legal or ethical restrictions on sharing data publicly. For information on unacceptable data access restrictions, please see http://journals.plos.org/plosone/s/data-availability#loc-unacceptable-data-access-restrictions.*

*In your revised cover letter, please address the following prompts:*

*a)* *If there are ethical or legal restrictions on sharing a de-identified data set, please explain them in detail (e.g., data contain potentially identifying or sensitive patient information) and who has imposed them (e.g., an ethics committee). Please also provide contact information for a data access committee, ethics committee, or other institutional body to which data requests may be sent.*

*b) If there are no restrictions, please upload the minimal anonymized data set necessary to replicate your study findings as either Supporting Information files or to a stable, public repository and provide us with the relevant URLs, DOIs, or accession numbers. Please see http://www.bmj.com/content/340/bmj.c181.long for guidelines on how to de-identify and prepare clinical data for publication. For a list of acceptable repositories, please see http://journals.plos.org/plosone/s/data-availability#loc-recommended-repositories.*

*We will update your Data Availability statement on your behalf to reflect the information you provide.*

Response 3: As suggested, we have addressed the prompts about the data request in our revised cover letter.

*4. Thank you for stating the following financial disclosure: 'No'*

*At this time, please address the following queries:*

*Please clarify the sources of funding (financial or material support) for your study. List the grants or organizations that supported your study, including funding received from your institution.*

*State what role the funders took in the study. If the funders had no role in your study, please state: “The funders had no role in study design, data collection and analysis, decision to publish, or preparation of the manuscript.”*

*If any authors received a salary from any of your funders, please state which authors and which funders.*

*If you did not receive any funding for this study, please state: “The authors received no specific funding for this work.”*

*Please include your amended statements within your cover letter; we will change the online submission form on your behalf.*

Response 3: We have amended the statement in our revised cover letter: “The authors received no specific funding for this work.”

*5. Thank you for stating the following in the Competing Interests section: 'No'*

*We note that one or more of the authors are employed by a commercial company, GE Healthcare.*

*a. Please provide an amended Funding Statement declaring this commercial affiliation, as well as a statement regarding the Role of Funders in your study. If the funding organization did not play a role in the study design, data collection and analysis, decision to publish, or preparation of the manuscript and only provided financial support in the form of authors' salaries and/or research materials, please review your statements relating to the author contributions, and ensure you have specifically and accurately indicated the role(s) that these authors had in your study. You can update author roles in the Author Contributions section of the online submission form.*

*Please also include the following statement within your amended Funding Statement.*

*“The funder provided support in the form of salaries for authors [insert relevant initials], but did not have any additional role in the study design, data collection and analysis, decision to publish, or preparation of the manuscript. The specific roles of these authors are articulated in the ‘author contributions’ section.”*

*If your* *commercial affiliation did play a role in your study, please state and explain this role within your updated Funding Statement.*

*b. Please also provide an updated Competing Interests Statement declaring this commercial affiliation along with any other relevant declarations relating to employment, consultancy, patents, products in development, or marketed products, etc.*

*Within your Competing Interests Statement, please confirm that this commercial affiliation does not alter your adherence to all PLOS ONE policies on sharing data and materials by including the following statement: "This does not alter our adherence to PLOS ONE policies on sharing data and materials.” (as detailed online in our guide for authors http://journals.plos.org/plosone/s/competing-interests). If this adherence statement is not accurate and there are restrictions on sharing of data and/or materials, please state these. Please note that we cannot proceed with consideration of your article until this information has been declared.*

*c. Please include both an updated Funding Statement and Competing Interests Statement in your cover letter. We will change the online submission form on your behalf.*

*Please know it is PLOS ONE policy for corresponding authors to declare, on behalf of all authors, all potential competing interests for the purposes of transparency. PLOS defines a competing interest as anything that interferes with, or could reasonably be perceived as interfering with, the full and objective presentation, peer review, editorial decision-making, or publication of research or non-research articles submitted to one of the journals. Competing interests can be financial or non-financial, professional, or personal. Competing interests can arise in relationship to an organization or another person. Please follow this link to our website for more details on competing interests:* [*http://journals.plos.org/plosone/s/competing-interests*](http://journals.plos.org/plosone/s/competing-interests)

Response 3: The commercial affiliation did not play any role in our study. We have decided to remove the only author employed by the commercial company (GE Healthcare) from the author list. Therefore, there are no changes in our “Funding Statement” and “Competing Interests Statement” in the revised manuscript.

*6. Please include your tables as part of your main manuscript and remove the individual files. Please note that supplementary tables should be uploaded as separate "supporting information" files.*

Response 3: As suggested, we have included the tables as part of the main manuscript in the revised manuscript and have removed the individual table files.
